# *Spongionella* Secondary Metabolites Protect Mitochondrial Function in Cortical Neurons against Oxidative Stress

**DOI:** 10.3390/md12020700

**Published:** 2014-01-27

**Authors:** Marta Leirós, Jon A. Sánchez, Eva Alonso, Mostafa E. Rateb, Wael E. Houssen, Rainer Ebel, Marcel Jaspars, Amparo Alfonso, Luis M. Botana

**Affiliations:** 1 Departamento de Farmacología, Facultad de Veterinaria, Universidad de Santiago de Compostela, Lugo 27003, Spain; E-Mails: marta.leiros@usc.es (M.L.); jonandoni.sanchez@rai.usc.es (J.A.S.); eva.alonso@usc.es (E.A.); amparo.alfonso@usc.es (A.A.); 2 Marine Biodiscovery Centre, Department of Chemistry, University of Aberdeen, Meston Walk, Aberdeen, Scotland AB24 3UE, UK; E-Mails: mostafa19772002@yahoo.com (M.E.R.); w.houssen@abdn.ac.uk (W.E.H.); r.ebel@abdn.ac.uk (R.E.); m.jaspars@abdn.ac.uk (M.J.); 3 Pharmacognosy Department, Faculty of Pharmacy, Beni-Suef University, Beni-Suef 32514, Egypt; 4 Institute of Medical Sciences, University of Aberdeen, Ashgrove Road West, Aberdeen, Scotland AB25 2ZD, UK

**Keywords:** diterpenes, *Porifera*, mitochondrial function, oxidative stress, neurodegenerative disorders

## Abstract

The marine habitat provides a large number of structurally-diverse bioactive compounds for drug development. Marine sponges have been studied over many years and are found to be a rich source of these bioactive chemicals. This study is focused on the evaluation of the activity of six diterpene derivatives isolated from *Spongionella* sp. on mitochondrial function using an oxidative *in vitro* stress model. The test compounds include the Gracilins (A, H, K, J and L) and tetrahydroaplysulphurin-1. Compounds were co-incubated with hydrogen peroxide for 12 hours to determine their protective capacities and their effect on markers of apoptosis and Nrf2/ARE pathways was evaluated. Results conclude that Gracilins preserve neurons against oxidative damage, and that in particular, tetrahydroaplysulphurin-1 shows a complete neuroprotective activity. Oxidative stress is linked to mitochondrial dysfunction and consequently to neurodegenerative disorders like Parkinson and Alzheimer diseases, Friedreich ataxia or Amyotrophic lateral sclerosis. This neuroprotection against oxidation conditions suggest that these metabolites could be interesting lead candidates in drug development for neurodegenerative diseases.

## 1. Introduction

Sponges are the animals of the phylum *Porifera*. They are multicellular invertebrates dating back 700-800 million years [1]. Their survival and wide distribution are due to their ability to adapt to environmental changes and competitiveness with the biota [2]. Sponges are aquatic metazoa with a simple structural organization consisting of a network of canals. The water penetrates through their external pores, allowing their internal circulation and the filtration of organic particles and microorganisms [1].

**Figure 1:**
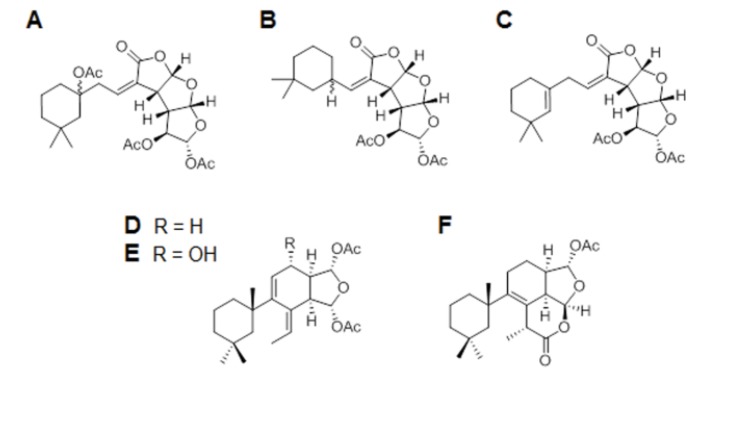
*Spongionella* compound structures: Gracilin J (**A**); Gracilin K (**B**); Gracilin H (**C**); Gracilin A (**D**); Gracilin L (**E**); and Tetrahydroaplysulphurin-1 (**F**).

Marine sponges are a huge source of potent compounds [3,4,5]. The majority of these secondary metabolites are generated for a defensive purpose [4,5], for example, to protect the organism against pathogenic bacteria, algae, fungi and other potential predators [4], so they are likely to be biologically active compounds. These bioactive products are possible candidates for drug development [3,4,5,6], and consequently with the original defense activity of these compounds, they show anti-fungal, anti-inflammatory [6], anti-cancer [5,6], antiviral [4] and antioxidant [7] effects. On various occasions, they have been described as neuroprotectors due to their ability to up-regulate antioxidant enzymes that lead to a decrease in the generation and accumulation of reactive oxygen species (ROS). In addition, the modulation of intracellular signal transduction molecules reduces oxidative stress and inflammation and consequently, restores the mitochondrial function [8]. Some of these metabolites with *Porifera* origin act through different signaling pathways [9] and kinases involved in neurodegenerative diseases [10], becoming an interesting group of compounds for pharmacological drug development. The genus *Spongionella* has contributed with several metabolites, such as diterpenes, nor- and bisnorditerpenes, furanosesterterpenes and polyhydroxylated sterols (e.g., Figure 1). Among these compounds were isolated Gracilins from *Spongionella gracilis* which are considered an interesting group of compounds because of their structures and effects towards tyrosine kinases [11].

Neurons are responsible for a high percentage of total oxygen body consumption and therefore they are more sensible to oxygen toxicity [12,13]. ROS overproduction leads to oxidative damage and this is related with neurodegenerative diseases [14]. Oxidative stress is an imbalance between ROS generation and antioxidants levels. As a result of the ROS increase, cell damage occurs in form of protein, DNA and RNA oxidation and lipid peroxidation [12]. The main ROS are superoxide (O_2_^•−^), hydrogen peroxide (H_2_O_2_) and the hydroxyl radical (OH^•^) whereas the principal antioxidant defenses are the enzymes superoxide dismutase (SOD), glutathione peroxidase (GPx), catalase (CAT) and the non-enzymatic antioxidant glutathione (GSH) that promote the conversion of H_2_O_2_ to H_2_O [14] by scavenging free radicals or working as a co-substrate of GPx [15]. The mitochondria have specific antioxidant protection, including the manganese SOD enzyme, located in the mitochondrial matrix, and the copper/zinc SOD, placed in the mitochondrial inter-membrane space but also in the cytosol [16]. The nuclear factor E2-related factor 2 (Nrf2) is the principal regulator of the genes that lead to the antioxidant response elements (AREs). In non-oxidative stress conditions, Nrf2 is located in the cytosol attached to Kealch-like ECH associated protein 1 (Keap1), forming the Nrf2-Keap1 complex. This complex is linked to the phosphoglycerate mutase 5 (PGAM5), a protein located in the outer mitochondrial membrane, and to the actin filament network. However, in oxidative stress, the actin-Nrf2-Keap1 complex is destabilized by ROS allowing the liberation and phosphorylation of Nrf2, and thus its translocation to the nucleus, where it activates the ARE response and consequently the antioxidant cell protection [17].

The high energy demand in neurons is provided by mitochondria, due to the ATP production in the electron transport chain. Occasionally, some electrons are liberated from the electron transport chain and react with molecular oxygen, producing ROS and hence oxidative stress [18], that leads to the apoptosis process. The apoptosis intrinsic pathways are directly related to mitochondria and they are activated by several stress conditions that lead to outer mitochondrial membrane permeabilization and the consequent release of cytochrome C (Cyt C). This protein is going to be trafficked to the apoptosome through its association with the apoptotic protease-activating factor (Apaf1) and deoxyadenosine triphosphate (dATP). This junction induces caspase-9 activation and the initiation of the caspase cascade. Moreover, caspase-8, activated on the extrinsic apoptotic pathway, promotes Cyt C release and cleavages the procaspase-3, producing the caspase-3 active form able to break down different substrates and provoke cell death [19]. Moreover, ROS induce the elevation of cytosolic Ca^2+^ levels that results in entry into the mitochondria. Because this mitochondrial overload occurs, the prolonged mitochondrial permeability transition pore (mPTP) is opened [12]. The mPTP is composed of several elements, cyclophylin D (cypD), voltage dependent anion channel (VDAC), and the adenine nucleotide translocase (ANT); the anion fluxes that are strongly influenced by oxidative stress due to these components are ROS targets. As a consequence, the mitochondrial membrane potential (ΔΨm) diminishes and pro-apoptotic factors—as Bax, Bad and cytochrome c—are released to the cytosol, resulting in an increased ROS production. The signaling pathway of mitogen-activated protein kinases (MAPK) like p38 and Jun *N*-terminal protein kinase (JNK) are activated by ROS and are involved in apoptosis and inflammatory processes [20].

Oxidative stress is associated with mitochondrial dysfunction and is a common feature in neurodegenerative diseases, while, on the other hand, natural products obtained from sponges have demonstrated antioxidant activity and neuroprotective abilities. These two facts have led us to study the effect of these six natural diterpenes isolated from *Spongionella* sp. on mitochondrial function in cortical neurons under oxidative stress conditions, their capacity to regulate the antioxidant Nrf2/ARE pathway, inhibition of apoptosis and their possible future application to drug development for neurodegenerative disorders.

## 2. Results and Discussion

### 2.1. *Spongionella* Compound Effects on Cortical Neurons Viability

We selected the MTT assay as the cell viability test because of its smooth correspondence between the cytotoxicity of the compounds and its decrease in mitochondria activity in neurons [21]. The effect of compounds over the viability of cortical neurons was studied at several concentrations (0.01, 0.05, 0.1 and 1 µM) for 48 h and no cytotoxicity was observed (data not shown).

### 2.2. *Spongionella* Compounds Show Neuroprotection Activity against H_2_O_2_ Insults

**Figure 2:**
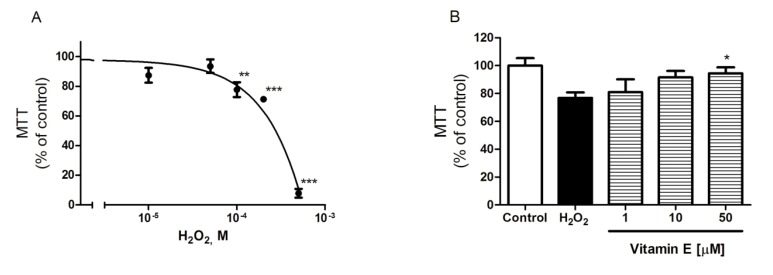
Oxidative stress *in vitro* model. (**a**) H_2_O_2_ induced cytotoxicity in primary cortical neurons after 12 h of treatment assayed by MTT. Data are presented in percentage respect non-treated control; (**b**) Protection of neuronal cells against 200 µM H_2_O_2_ by the antioxidant Vitamin E. Results are presented in percentage respect non-treated control and compared to cells treated with 200 µM H_2_O_2_. Data are mean ± SEM of three independent experiments performed by triplicate. Values are compared to the H_2_O_2_ positive control by ANOVA statistical analysis followed by post hoc Dunnett’s *t* test. * (*p* < 0.05), ** (*p* < 0.01) and *** (*p* < 0.001).

In order to study the influence of natural compounds over mitochondrial activity in an oxidative stress *in vitro* model, H_2_O_2_ was used as oxidizing agent. Direct insults of H_2_O_2_ have been described for oxidative stress studies [22] and neurodegenerative diseases in *in vitro* models, such as in the Parkinson disease model [23]. Cortical neurons were incubated for 12 h with H_2_O_2_ at 10, 50, 100, 200 and 500 µM and cell viability was determined by MTT assay. As can be seen in Figure 2A, H_2_O_2_ produced a cytotoxic dose-dependent effect causing complete cell death at 500 µM (92.1% ± 3.0%). Moreover, 200 µM induced a 28.7% ± 1.1% viability decrease with respect to control that opened a wide enough window in the oxidative stress response in mitochondria to be able to study the effects of the compounds without inducing apoptosis or reaching necrosis. Following the *in vitro* model development, Vitamin E was used to validate it. Vitamin E, as an antioxidant compound, is an important ROS scavenger with neuroprotective effects [24]. It was co-incubated at 1, 10 and 50 µM with H_2_O_2_ at 200 µM for 12 h and finally the viability was assayed by MTT. Results, represented in Figure 2B, demonstrate a dose-dependent increase of viability, reaching control levels with 50 µM Vitamin E treatment (94.4% ± 4.4%, *p* < 0.05) and confirming the oxidative stress in the *in vitro* cellular model.

**Figure 3:**
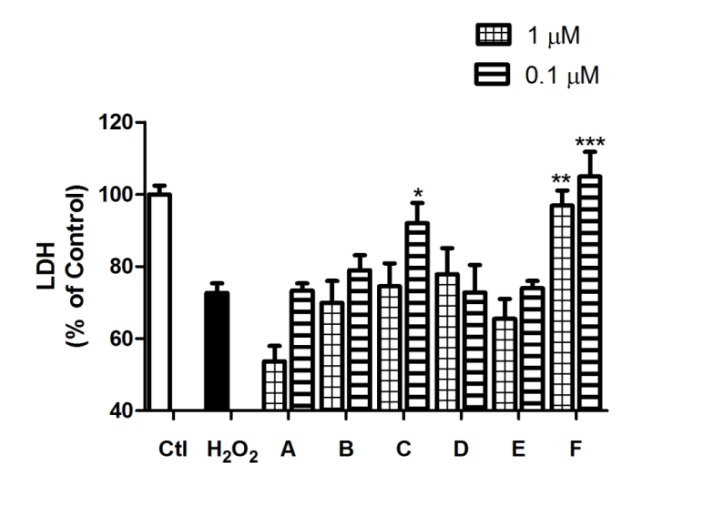
Compounds C and F protect neurons membrane integrity in oxidative stress conditions. Cell membrane integrity was studied by LDH assay, and cellular viability was increased in neurons treated with C and F compounds. Cortical neurons were co-incubated with 200 µM H_2_O_2_ and compounds at 1 and 0.1 µM. Compounds C and F, represented in the graph, were the only ones that were able to avoid LDH release. All values are shown in percentage in respect to the non-treated control (Ctl) and compared to cells treated with 200 µM H_2_O_2_ by ANOVA statistical analysis followed by post hoc Dunnett’s *t* test. * (*p* < 0.05), ** (*p* < 0.01) and *** (*p* < 0.001). Data are mean ± SEM of three or more independent experiments performed by triplicate.

**Figure 4:**
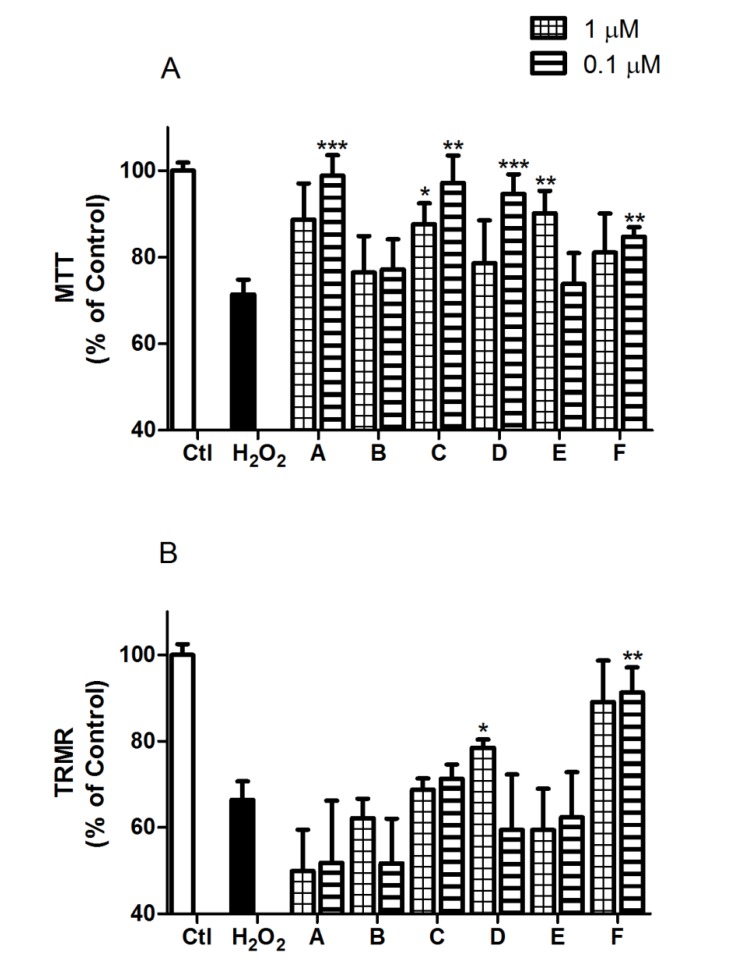
*Spongionella* compounds effects over mitochondrial function and ∆Ψm in an oxidative stress situation. (**a**) Mitochondrial function was evaluated by MTT, and cellular viability was augmented in cortical neurons incubated with A, C, D, E and F compounds; (**b**) TRMR assay was carried out to analyze the ∆Ψm, and compounds D and F maintained the membrane potential. All values are shown in percentage with respect to the non-treated control and compared to cells treated with 200 µM H_2_O_2_ by ANOVA statistical analysis followed by post hoc Dunnett’s *t* test. * (*p* < 0.05), ** (*p* < 0.01) and *** (*p* < 0.001). Data are mean ± SEM of three or more independent experiments performed by triplicate.

The neuroprotective effects of *Spongionella* compounds in oxidative stress conditions were studied using this model by analyzing two viability parameters: LDH levels and MTT assay. LDH is a cytoplasmic enzyme that in case of cell membrane damage it is released to the culture medium, being a cell membrane integrity indicator and correlating with cell viability. Otherwise, MTT assay determines the mitochondrial function activity [25], well associated with neurons’ survival measurements [21]. Compounds at two different concentrations (0.1 and 1 µM) were co-incubated with H_2_O_2_ (200 µM) for 12 h, and viability assays were performed. LDH assay results showed that cells treated with 200 µM H_2_O_2_ have a viability decrease of 27.3% ± 2.6% (*p* < 0.001), and only two compounds were demonstrated to protect neurons at membrane level reaching basal measurements. Compound C gave a viability of 92.0% ± 5.6% (*p* < 0.05) at 0.1 µM and compound F a 96.9% ± 4.2% (*p* < 0.01) at 1 µM and a 104.9% ± 6.9% (*p* < 0.001) at 0.1 µM (Figure 3). MTT results are present in Figure 4A and they showed more activity than in LDH assay. Five of the six compounds tested present neuroprotection effects at mitochondrial function level. The response of neurons treated with 200 µM H_2_O_2_ was a decrease on mitochondrial activity of 28.6% ± 3.4% (*p* < 0.001) and all compounds except B diminished this percentage, achieving control levels with 0.1 µM treatment of compounds A (98.9% ± 4.7%, *p* < 0.001), C (97.2% ± 6.3%, *p* < 0.01) and D (94.6% ± 4.6%, *p* < 0.001).

∆Ψm investigation provides new data to understand the activity of these compounds against H_2_O_2_ insults. The TRMR assay shows a decrease of 33.6% ± 4.3% (*p* < 0.001) in ∆Ψm in cells incubated with 200 µM H_2_O_2_ for 12 h. Carrying out the same treatments as in viability assays, *Spongionella* compounds were co-incubated with H_2_O_2_, and the TRMR test was accomplished. Compounds D and F increased the ∆Ψm with respect to neurons treated with H_2_O_2_ (Figure 4B), being the most effective compound F at 0.1 µM (91.3% ± 5.8%, *p* < 0.01).

**Figure 5:**
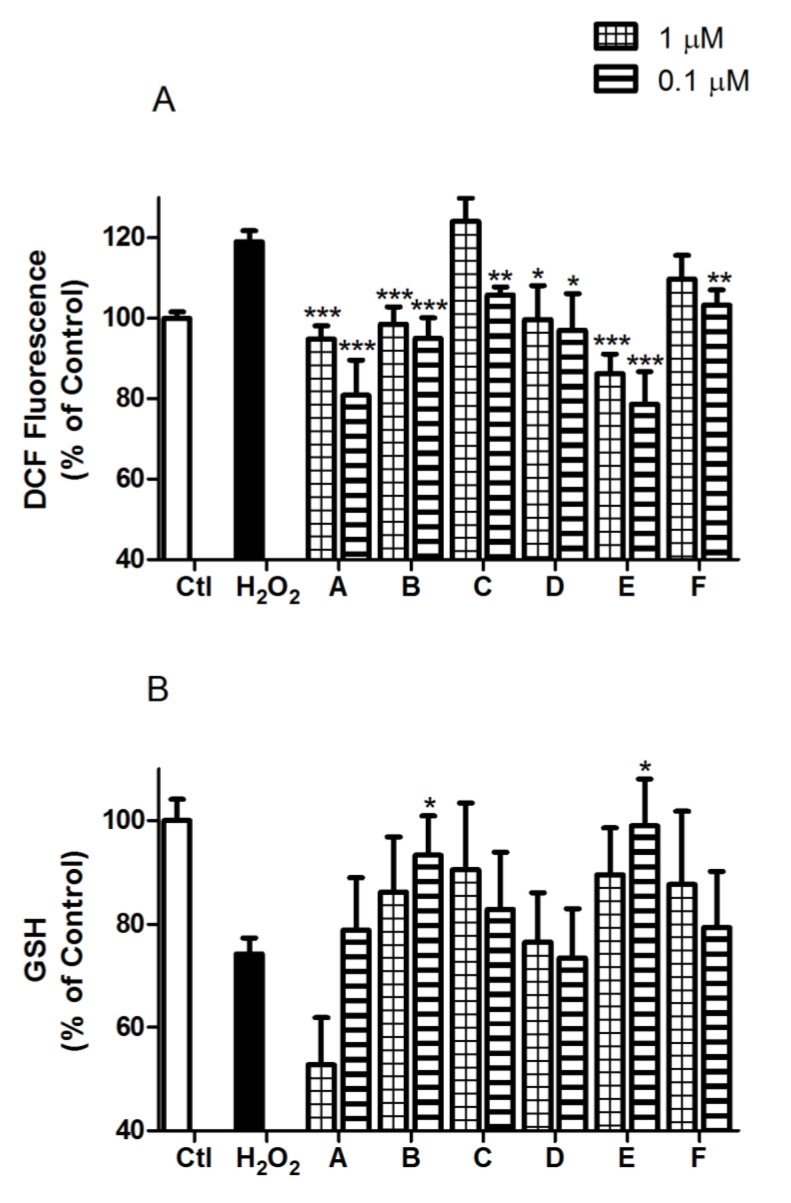
*Spongionella* compounds preserves ROS and GSH levels in oxidative stress conditions. (**a**) ROS production was analyzed with the DCFH-DA reaction and all compounds reduced ROS with respect to the stress control; (**b**) GSH was determined by ThiolTracker™ Violet dye, maintaining the basal levels in neurons treated with compounds B and E. In both assays, ROS and GSH, compounds were co-incubated with 200 µM H_2_O_2_ at 1 and 0.1 µM. Data are expressed in percentage with respect to non-treated control and compared to cells treated with 200 µM H_2_O_2_ by ANOVA statistical analysis followed by post hoc Dunnett’s *t* test. * (*p* < 0.05), ** (*p* < 0.01) and *** (*p* < 0.001). Values are mean ± SEM of three or more independent experiments performed by triplicate.

Mitochondria are the main producers of endogenous ROS by oxidative phosphorylation in the electron transport chain, and in oxidative stress conditions, it is observed that its production increases. Accordingly, cortical neurons treated with H_2_O_2_ for 12 h increase ROS levels in a 19.0% ± 2.7% (*p* < 0.001). Co-incubations of the natural compounds with H_2_O_2_ were done, as previously described and ROS production was measured. The six compounds demonstrate the ability to diminish ROS compared to cells treated with H_2_O_2_ alone (Figure 5A). The decrease was significant for all the compounds tested, except for C and F at 1 µM. The reduction of ROS production was more pronounced at 0.1 µM in all cases.

To complete the study of the effects produced by *Spongionella* compounds on mitochondria scope, their influence on GSH was studied. GSH is one of the antioxidant mitochondrial systems of protection against oxidative damage. The 12 h H_2_O_2_ treatment produces a GSH level decrease of 25.8% ± 3.1% (*p* < 0.001) in primary cortical neurons. Once again cells were incubated with the natural compounds and H_2_O_2_ as detailed above. Their effects over GSH in an oxidative stress environment were determined and represented in Figure 5B. Treatments of compounds B and E at 0.1 µM and H_2_O_2_ produce a significant increase in GSH in relation to neurons exclusively incubated with H_2_O_2_. The most effective was compound E achieving a 99.0% ± 9.0% (*p* < 0.05) that represents the complete reestablishment of GSH control levels.

### 2.3. Variations on [Ca^2+^]c Induced by *Spongionella* Compounds

**Figure 6:**
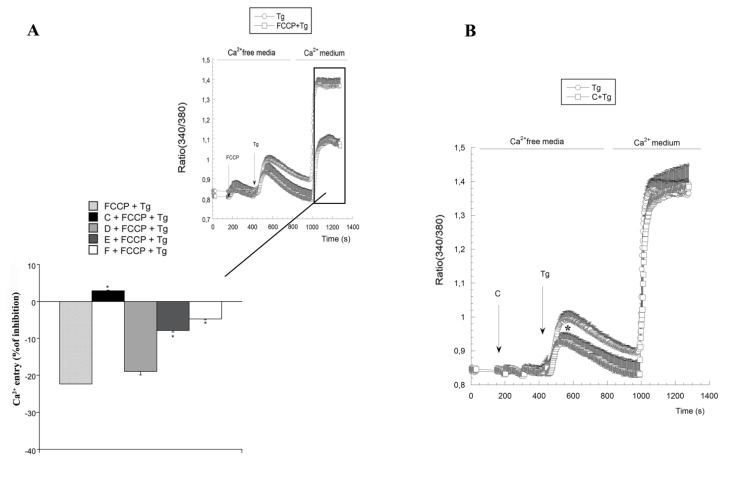
Blockage of FCCP inhibition on cytoplasmic calcium entrance induced after endoplasmic reticulum emptying by Thapsigargin (Tg) produced by *Spongionella* compounds. (**a**) Bars histogram represents maximal Ca^2+^ entry observed after the ion restoration. The potency of these compounds to block the effect of FCCP calcium entrance induced by Tg is C > E > F > D; (**b**) Compound C effect over Ca^2+^ release from endoplasmatic reticulum induced by thapsigargin. * Significant differences between FCCP and compounds C, D, E and F pre-treated cells.

The four most promising compounds (C to F) were selected for the evaluation of [Ca^2+^]c. A neuroblastoma cell line was used to perform this assay and cells were loaded with 2.5 μM Fura-2AM, used as cellular calcium fluorescent marker. Mitochondria play an important role in several cellular routes providing the main part of the energy required by cells through oxidative phosphorylation. It is observed that this organelle has a key role in Ca^2+^ signaling, apoptosis and aging and that therefore, it is related with neurodegenerative diseases. It is known that thapsigargin, a sarcoplasmic/endoplasmic reticulum calcium dependent ATP-ase (SERCA) blocker, depletes intracellular Ca^2+^ stores in Ca^2+^ free medium and thus the opening of calcium release operated channels (CRAC channels) in the plasma membrane, producing a fast cytosolic Ca^2+^ increase when the ion is restored to the extracellular medium [26,27]. This Ca^2+^ increase is diminished in the presence of the oxidative phosphorylation uncoupler Carbonyl cyanide 4-(trifluoromethoxy)phenylhydrazone (FCCP). This protonophore stimulates mitochondrial respiration, increases O_2_ consumption and collapses the proton gradient across the mitochondrial inner membrane and therefore alters the electron transport chain enhancing oxidation, ROS production and reducing Ψm [28]. We use FCCP to study if the highlighted compounds interfere with FCCP effects over mitochondria. Cells were incubated with the compounds C to F at 1 µM and after that FCCP and thapsigargin were added. In Figure 6A, we observe that FCCP blocks Ca^2+^ entry by more than 60% and that this effect is completely inhibited in the presence of compound C, which even increases Ca^2+^ entry. Compounds E and F are able to inhibit FCCP effect by a 50%, whereas compound D is the least effective with a minimal inhibition. These results point to a direct interaction of these compounds with the mitochondrial oxidative phosphorylation. Moreover, compound C was the only one that produces a small decrease of Ca^2+^ endoplasmatic reticulum release induced by thapsigargin (Figure 6B).

### 2.4. *Spongionella* Compounds Inhibit Caspase-3

**Figure 7:**
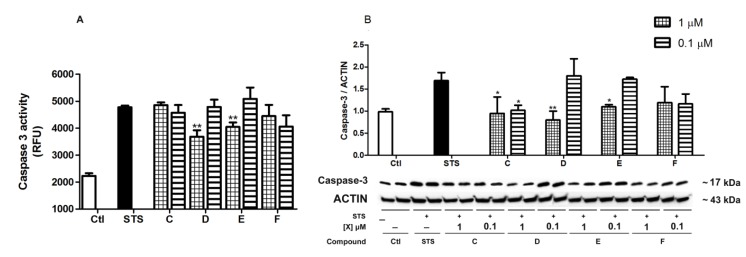
Effects of *Spongionella* compounds on the inhibition of caspase-3 activity (**a**) and expression (**b**), used as an apoptosis marker. Co-incubations of 0.5 µM STS and compounds at 1 and 0.1 µM were performed and then caspase-3 activity and cytosolic levels were measured. Compounds D and E at 1 µM inhibit this activity and expression compared to neurons only treated with STS. Activity values are shown in RFU and expression data are in ratio caspase-3/actin, and both are compared to cells treated with 0.5 µM STS. Results were statistically analyzed by ANOVA followed by post hoc Dunnett’s *t* test *versus* STS treatments, * (*p* < 0.05) and ** (*p* < 0.01). Data are mean ± SEM of three or more independent experiments.

The loss of calcium homeostasis leads to mitochondrial dysfunction and finally to the activation of apoptosis pathways through the cytochrome c release and caspase activation [29]. The positive calcium results prompted us to further investigate caspase activity. To induce the activation of caspase-3, and hence apoptosis, cortical neurons were incubated with STS, a non-specific inhibitor of protein kinase C (PKC). Because of the spontaneous increased caspase-3 activity in neurons *in vitro*, experiments were performed on three days *in vitro* with STS 0.5 µM [30]. Results are presented in Figure 7A, expressed in relative fluorescence units (RFU). The caspase-3 activity was doubled after 6 h incubation with STS (4633.2 ± 187.7 RFU) compared to control neurons (2179.8 ± 124.4 RFU, *p* < 0.001). Neurons were co-treated with STS and compounds C to F at 1 and 0.1 µM for 6 h. As can be observed in Figure 7A, compounds D (3882.3 ± 265.3 RFU, *p* < 0.01) and E (4044.1 ± 165.9 RFU, *p* < 0.01) inhibit significantly caspase-3 enzymatic activity at 1 µM with respect to neurons only treated with STS. Furthermore, the expression of caspase-3 cleavage was analyzed by western blotting after 6 h treatments and results were plotted as caspase-3/actin ratio (Figure 7B). STS treated neurons presented an increase expression of caspase-3 cleavage *versus* control neurons. Confirming the activity results, compounds D and E at 1 µM reduced caspase-3 cytosolic levels respect to STS treated cells. However, as can be seen in Figure 7B, compound C also produced a significant diminish on caspase-3 expression at both concentrations 1 and 0.1 µM that was not observed with the caspase-3 activity kit.

### 2.5. Nrf2 Translocation Induced by *Spongionella* Compounds

**Figure 8:**
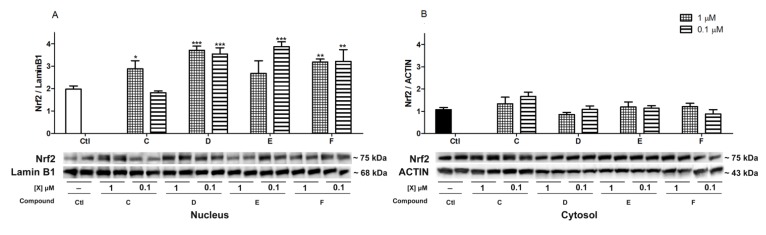
Induction of Nrf2 translocation by *Spongionella* compounds. Nrf2 levels were measured in lysates of neurons incubated with the compounds for 6 h. (**a**) Nucleus and (**b**) cytosolic results are presented separated. Nrf2 was normalized with Lamin B1 for nucleus samples and Actin for cytosolic lysates and bars represent the ratio. Results were statistically analyzed by ANOVA followed by post hoc Dunnett’s *t* test, compared to their respective controls. * (*p* < 0.05) and ** (*p* < 0.01). Data are mean ± SEM of three or more independent experiments.

A key molecule in the cellular antioxidant response is the transcriptional factor Nrf2 for the ARE pathway. This antioxidant response includes a diverse group of genes that encode enzymes like superoxide dismutase (SOD), catalase (CAT), glutathione peroxidase (GPx) or glutathione reductase (GR) among others, and all of them are involved in oxidative stress protection [31,32]. When this factor is activated, it is translocated to the cellular nucleus, so we studied the expression of Nrf2 in the nucleus as a marker of its activation. In order to evaluate Nrf2 levels in the nucleus and cytosol of cortical neurons, cells were treated at 1 and 0.1 µM with the compounds selected, C to F. After 6 h of incubation, cell lysates were obtained and processed to analyze Nrf2 expression by western blot. Results are expressed in ratio of Nrf2/Lamin B1 for nuclear lysates and Nrf2/Actin for cytosolic samples (Figure 8). All compounds produce an increase expression of Nrf2 in the nucleus. This augmentation of Nrf2 in neurons treated with 0.1 µM is higher than in cells treated with 1 µM for compounds D, E and F. While compound C produces a significant increase of Nrf2 in the nucleus at the higher concentration, no variations were observed at 0.1 µM. In cytosol Nrf2 level does not significantly change with respect to the control for any of the compounds. These cytosol data correlate with the nucleus results obtained and a consequent translocation of Nrf2.

Marine natural products are becoming an important source in drug development. The great sponge biodiversity and the structurally diverse secondary metabolites they produce open a wide range of possible research areas. These natural compounds are especially effective as anti-tumor potential drugs [5,6], but also show anti-fungal, anti-inflammatory [6], antiviral [4] and antioxidant [7] effects. In this study, compounds from Gracilin family (A–E) and tetrahydroaplysulphurin-1 (F), isolated from *Spongionella* sp., were evaluated in neurons under conditions of oxidative stress induced by H_2_O_2_, resulting in neuroprotective effects. Neurons are one of the weakest populations against oxidative processes, which makes them vulnerable in increased oxidative stress conditions and therefore are an important factor in neurodegenerative pathologies such as Alzheimer disease. In this research, we use an *in vitro* stress oxidation model obtained from primary cortical neurons from mice to mimic the pathological conditions in the presence of free radicals in the brain. Mitochondrial respiration produces ROS that in physiological conditions is scavenged by the antioxidant defenses. However, when these defenses fail ROS accumulation is followed by a cascade of mitochondrial failures that end in apoptosis and cellular death. In the present work we report that, except for compound B, *Spongionella* compounds are active metabolites against oxidative stress damage in primary cortical neurons, exhibiting mitochondrial function protection and a reduction of ROS production. Additionally, compounds C and F are the only ones that showed the ability of preserving cell membrane integrity by LDH assay. Three of the Gracilins tested (Gracilin H, Gracilin A and Gracilin L, named as compound C, D and E in this work) and the tetrahydroaplysulphurin-1 (compound F) showed a complete neuroprotective effect in primary neurons with a restored mitochondrial membrane potential and an inhibition of the alterations produced by the mitochondrial uncoupler FCCP. Furthermore, three of these compounds (Gracilin A and L and tetrahydroaplysulphurin-1) elicited an enhanced expression of Nrf2 in the nucleus which is related with the antioxidant pathway activation. However, only two compounds (Gracilin A and L) were able to reduce the caspase-3 activity induced by STS which could be explained because of the compounds’ structures, differentiated exclusively by one radical. These compounds’ ability was verified by their capacity to reduce caspase-3 cleavage expression. Nevertheless, Gracilin H produces no effect on its activity but decreases the cytosolic levels that can be explained by a different sensitivity to the techniques employed.

The high activity of these compounds against oxidative stress can be due to several reasons. In this research, we show the ability of these compounds to increase Nrf2 levels in the nucleus and consequently the antioxidant pathway is activated. However, as it has been previously reported, their capacity to inhibit the epidermal growth factor receptor (EGFR) tyrosine kinase—D to F being the most potent compounds [11]—supports the antioxidant activity presented in this work. EGFR is a tyrosine kinase cell surface receptor, involved in the cell proliferation and apoptosis regulation, and the inhibitors of this HER/ErbB family member have been described as potential anti-cancer drugs [33]. Additionally, the compounds Gracilin A and aplysulphurin A are potential anti-inflammatory drugs due their capacity of phospholipase A_2_ (PLA_2_) inhibition, associated with a structure–function relationship [34]. Moreover, similar structures are possible targets for neurodegenerative diseases, due to the ability of PLA_2_ inhibitors to diminish amyloid β-induced ROS production [35], strengthening our results.

## 3. Experimental Section

### 3.1. Porifera Compounds Information

The Marine Biodiscovery Centre (Department of Chemistry, University of Aberdeen) supplied the library of compounds. The present investigation focused on six natural compounds isolated from *Spongionella* sp. (Table 1). Compounds were purified from their sponge sources which were freeze-dried, extracted with MeOH and MeOH/CH_2_Cl_2_ to obtain the crude extract. The crude extract of each organism was dissolved in H_2_O and passed through Diaion HP20 resin and re-concentrated under vacuum to obtain a salt-free extract. This extract was subjected to multiple steps of liquid/liquid fractionation, SiO_2_, Sephadex LH-20 and RP-C18 chromatography to obtain the pure compounds. The structure elucidation of these compounds was based on their HRESIMS analysis as well as direct comparison with the previously reported NMR spectral data [11,36]. Compounds tested are compounds A to F (Figure 1).

**Table 1:** *Spongionella* compounds information.

Code	Chemical Name	Formula	MW	Source Organism
**A**	Gracilin J	C_24_H_32_O_10_	480.19	*Spongionella* sp.
**B**	Gracilin K	C_21_H_28_O_8_	408.17	*Spongionella* sp.
**C**	Gracilin H	C_22_H_28_O_8_	420.17	*Spongionella* sp.
**D**	Gracilin A	C_23_H_34_O_5_	390.24	*Spongionella* sp.
**E**	Gracilin L	C_23_H_34_O_6_	406.23	*Spongionella* sp.
**F**	Tetrahydroaplysulphurin-1	C_22_H_32_O_5_	376.22	*Spongionella* sp.

### 3.2. Primary Cortical Neurons

Primary cortical neurons were obtained from embryonic day 15–18 mice fetuses as described [37]. All protocols described in this work were revised and authorized by the University of Santiago de Compostela Institutional animal care and use committee, and comply with European legislation on the use and management of experimental animals.

Swiss mice were used to obtain primary cultures of cortical neurons. Briefly, cerebral cortexes were removed and neuronal cells were dissociated by trypsinization at 37 °C, followed by mechanical trituration in DNase solution (0.005% w/v) with a soybean-trypsin inhibitor (0.05% w/v). Cells were suspended in DMEM supplemented with *p*-amino benzoate, insulin, penicillin and 10% fetal calf serum. The cell suspension was seeded in 12 multi-well plates with coverslips and 96 multi-well plates pre-coated with poly-d-lysine and incubated in a humidified 5% CO_2_/95% air atmosphere at 37 °C. Cytosine arabinoside 20 μM, was added before 48 h of culture to prevent growing of non-neuronal cells. Treatments were carried out in 3 day *in vitro* (*div*) for caspase-3 experiments, 4 *div* for Nrf2 measurements and 4–5 *div* for neuroprotection assays.

### 3.3. Neuroblastoma Cell Line

SH-SY5Y cells were purchased from ATCC Number CRL-2266. They were plated in 25 cm^2^ flask at a cultivation ratio of 1:10. The cells were maintained in Eagle’s Minimum Essential Medium (EMEM) from ATCC and F12 Medium (Invitrogen, Paisley, UK) in a proportion 1:1 supplemented with 10% fetal bovine serum (FBS) from PAA, 100 UI/mL penicillin and 100 µg/mL streptomycin. The neuroblastoma cells were dissociated weekly using 0.05% trypsin/EDTA (1×) (Invitrogen, Paisley, UK). All calcium experiments were carried out using the neuroblastoma cell line.

### 3.4. Chemicals and Solutions

Plastic tissue-culture dishes were purchased from Falcon (Madrid, Spain). Fetal calf serum was obtained from Gibco (Glasgow, UK) and Dulbecco’s Modified Eagle’s medium (DMEM) was from Biochrom (Berlin, Germany). Thapsigargin (TG) was from Alexis Corporation (Läufelfingen, Switzerland), Fura-2AM was obtained from Molecular Probes (Leiden, The Netherlands), carbonyl cyanide *p*-(trifluoromethoxy) (FCCP) and all other chemicals were reagent grade and purchased from Sigma-Aldrich (Madrid, Spain).

### 3.5. Cytotoxicity Assay

The MTT (3-[4,5-dimethylthiazol-2-yl]-2,5-diphenyltetrazoliumbromide) assay was the test selected to analyzed cell viability as previously described [38,39]. Primary cortical neurons were grown in 96 well plates and exposed to different compound concentrations (0.01, 0.05, 0.1 and 1 µM) added to the culture medium. Cultures were maintained in the presence of the pure compounds at 37 °C in humidified 5% CO_2_/95% air atmosphere for 48 h. Saponin was used as cellular death control and its absorbance was subtracted from the other data. After treatment time assay was performed and cells were rinsed and incubated for 1 h with a solution of MTT (500 µg/mL) dissolved in saline buffer. After washing off excess MTT, cells were disaggregated with 5% sodium dodecyl sulfate and the absorbance of the colored formazan salt was measured at 595 nm in a spectrophotometer plate reader.

### 3.6. Neuroprotection Assays

Treatments were carried out in 96-well plate, as co-incubations of H_2_O_2_ 200 µM and the compounds at 1 and 0.1 µM for 12 h on 4–5 *div* neurons.

#### 3.6.1. Mitochondrial Function and ∆Ψm (Membrane Potential Variation) Assays

Mitochondrial function was studied by MTT test following the method previously described and variations in ∆Ψm were determined with the tetramethylrhodamine methyl ester (TMRM) assay [40]. For TMRM assays, cells were washed twice with saline solution and incubated with 1 µM TMRM for 30 min. Then cells were solubilized with 50% DMSO/water. Fluorescence values were obtained using a spectrophotometer plate reader (535 nm excitation, 590 nm emission).

#### 3.6.2. Cell Survival Measurement

The *in vitro* Toxicology Assay Kit (TOX7, Sigma) was used for measuring its activity, following the commercial protocol. This kit use LDH release as an indicator of cell survival.

### 3.7. Evaluation of ROS Generation

ROS was analyzed by a fluorescence assay using 7′,2′-dichlorofluorescein diacetate (DCFH-DA), as previously described [41]. Briefly, DCFH-DA penetrates into the cell and it is de-esterified to the ionized free acid (DCFH). DCFH reacts with ROS resulting the fluorescent 7′,2′-dichlorofluorescein (DCF). Cells were treated following the protocol above, and after incubations neurons were rinsed with saline solution and then were loaded with 20 µM DCF-DA for 30 min at 37 °C. Cells were washed and kept at room temperature for 30 min to allow a complete de-esterification. The DCF signal was measured using a fluorescence plate reader where excitation was monitored at 475 nm and emission at 525 nm.

### 3.8. Estimation of Glutathione Levels

GSH is the main intracellular free thiol in cells, so we used ThiolTracker™ Violet dye to estimate their levels in our treated cells. Neurons were washed with phosphate buffer solution and loaded with 10 µM ThiolTracker™ Violet dye for 1 h at 37 °C. After incubation, neurons were rinsed once and fluorescence was read at 404 nm excitation and 526 nm emission.

### 3.9. Determination of the Cytosolic Calcium Concentration [Ca^2+^]c

For [Ca^2+^]c determination, cells were seeded onto 18 mm glass coverslips and used between 48 and 72 h after plating at a density of 120,000 cells/glass coverslip. Afterward, they were washed twice with saline solution supplemented with 0.1% bovine serum albumin (BSA). Physiological saline solution was composed by (mM): NaCl 119, Mg(SO_4_) 1.2, NaH_2_PO_4_ 1.2, NaHCO_3_ 22.85, KCl 5.94, CaCl_2_ 1. Glucose 1 g/L was added to the medium living and osmotic pressure of 290 mOsm/kg of H_2_O. In all the assays, the solutions were equilibrated with CO_2_ before used, adjusting the final pH between 7.2 and 7.4.

Neuroblastoma cells were loaded with the Ca^2+^-sensitive fluorescent dye Fura-2 acetoxymethyl ester (Fura-2AM; 2.5 μM) for 10 min at 37 °C and 300 rpm in a saline solution described above, plus 0.1% BSA. After incubation, the loaded cells were washed twice with calcium saline solution. The glass coverslips were inserted into a thermostatted chamber at 37 °C (Life Science Resources, Royston, Herts, UK) and cells were viewed with a Nikon Diaphot 200 microscope, equipped with epifluorescence optics (Nikon 40× immersion UV-Fluor objective, Life Sciences Resources, Royston, Herts, UK). The thermostatted chamber was used in the open bath configuration, and additions were made by removal and addition of fresh bathing solution. The experiments were carried out at least three times.

The [Ca^2+^]c ratio was obtained from the images collected by fluorescent equipment (Lambda-DG4, Sutter Instrument Company, Novato, CA, USA). The Light source was a xenon arc bulb, and the different wavelengths used were chosen with filters. The excitation wavelengths for Fura-2AM were 340 and 380 nm, with emission at 505 nm.

### 3.10. Measurement of Caspase-3 Activity and Expression

Treatments for these assays were carried out with cortical neurons on 3 *div* and the apoptosis was induced with 0.5 µM staurosporine (STS) during 6 h. Caspase-3 activity was measured by the EnzChek^®^ Caspase-3 Assay Kit. This kit provides a non-fluorescent bisamide substrate (Z-DEVD-R110, Sigma, St. Louis, MO, USA) which is transformed by caspase-3 first to a monoamide and then to the green-fluorescent rhodamine 110. Cell lysates were obtained and processed following the commercial protocol and fluorescence was read (excitation 496 nm; emission 520 nm). Moreover, caspase-3 expression was determined by western blot. Cells were washed with cold PBS after treatments and lysed with a scrapper in a 50 mM Tris-HCl, 150 mM NaCl, 1% Triton X-100, 1 mM EDTA buffer solution, containing protease and phosphatase inhibitor cocktail (ROCHE, Mannhein, Germany). Lysates were collected in eppendorfs and then centrifuged at 12,000 rpm at 4 °C for 20 min to separate the cytosolic fraction. Bradford assay was used to quantify total protein concentration. Cell lysates containing 10 μg of total protein were resolved in gel loading buffer and lysates were electrophoresed through a 10% sodium dodecyl sulfate polyacrylamide gel (BIORAD, Hercules, CA, USA) and transferred onto PVDF membranes (Millipore, Single Oak Drive, Temecula, CA, USA). The membrane blocking and antibody incubation was performed by Snap i.d protein detection system. The immunoreactive bands were detected using the Supersignal West Pico Chemiluminescent Substrate or Supersignal West Femto Maximum Sensitivity Substrate (Pierce, Rockford, IL, USA) and the Diversity 4 gel documentation and analysis system (Syngene, Cambridge, UK). Chemiluminescence was measured with the Diversity GeneSnap software (Syngene, Cambridge, UK). Caspase-3 was detected with the primary antibody anti-Caspase-3 (5:1000, Millipore, Single Oak Drive, Temecula, CA, USA) with rabbit secondary antibody and it was normalized by β-Actin (1:20,000, Millipore, Single Oak Drive, Temecula, CA, USA).

### 3.11. Nrf2 Analysis by Western Blotting

The Nrf2 in nucleus and cytosol were analyzed by western blot. Cortical neurons were incubated with compounds for 6 h and afterward they were washed twice with ice-cold PBS. Cells were homogenized for 15 min in an ice-cold cytosolic hypotonic buffer solution composed of 20 mM Tris-HCl pH 7.4, 10 mM NaCl and 3 mM MgCl_2_, containing a Roche complete phosphatase/protease inhibitors cocktail. Then cells were scrapped and finally centrifuged at 3000 rpm at 4 °C for 10 min to obtain the protein cytosolic fraction. The supernatant was collected and the pellet was resuspended for 30 min, vortexing in 10 min intervals, in an ice-cold nuclear extraction buffer containing 100 mM Tris pH 7.4, 2 mM Na_3_VO_4_, 100 mM NaCl, 1% Triton X-100, 1 mM EDTA, 10% glycerol, 1 mM EGTA, 0.1% SDS, 1 mM NaF, 0.5% deoxycholate, 20 mM Na_4_P_2_O_7_, 1 mM PMSF and a protease inhibitor cocktail. The resuspended pellet samples were centrifuged at 14,000 × *g* at 4 °C for 30 min. The supernatants were collected as protein nuclear fractions. Bradford, electrophoresis and western blotting assays were performed as previously described. Samples were tested containing 10 μg (nuclear fraction) and 20 μg (cytosolic fraction). Nrf2 was detected with the primary antibody anti-NF-E2-related factor 2 (1:1000, Millipore, Single Oak Drive, Temecula, CA, USA) with rabbit secondary antibody. Nrf2 signal was normalized by Lamin B1 (1:1000, ABCAM, Cambridge, UK) for nuclear samples and β-Actin (1:20,000, Millipore, Single Oak Drive, Temecula, CA, USA) for cytosolic lysates.

### 3.12. Statistical Analysis

All the results are expressed as means ± SEM of three or more experiments. Assays carried out on 96 well plates were performed by triplicate. Statistical comparison was performed by one way ANOVA with Dunnett’s post hoc analysis. *p* values <0.05 were considered statistically significant.

## 4. Conclusions

Marine natural compounds, and specifically those of sponge origin, offer a wide variety of compounds, many of them with antioxidant properties. Since neurodegenerative diseases are closely associated with oxidative stress, these compounds are interesting candidates for drug development. In this research, we highlight the neuroprotective capacity of Gracilins J, K, H, A and L and tetrahydroaplysulphurin-1 with a complete mitochondrial protection. These antioxidant capacities open up future research about the particular interactions of these compounds with kinases involved in apoptosis or in the Nrf2-Keap1-ARE signal pathway, clarifying their mechanism of action over Nrf2 levels in the nucleus. These activities would be highly interesting for the development of new drugs for neurodegenerative diseases, as Parkinson, Alzheimer, Friedreich ataxia or Amyotrophic lateral sclerosis.

## Abbreviations

ROS: reactive oxygen species
GSH: glutathione
